# Evaluation of the Storage Conditions and Type of Cork Stopper on the Quality of Bottled White Wines

**DOI:** 10.3390/molecules26010232

**Published:** 2021-01-05

**Authors:** María Consuelo Díaz-Maroto, Manuel López Viñas, Lourdes Marchante, María Elena Alañón, Ignacio Javier Díaz-Maroto, María Soledad Pérez-Coello

**Affiliations:** 1 Area of Food Technology, Faculty of Chemical Sciences and Technologies, Regional Institute for Applied Scientific Research (IRICA), University of Castilla-La Mancha, Avda. Camilo José Cela 10, 13071 Ciudad Real, Spain; manuel.lvinas@uclm.es (M.L.V.); soledad.perez@uclm.es (M.S.P.-C.); 2 Instituto Regional de Investigación y Desarrollo Agroalimentario y Forestal de Castilla La Mancha (IRIAF), IVICAM, 13700 Ciudad Real, Spain; lmarchantec@jccm.es; 3 Area of Food Technology, Higher Technical School of Agronomic Engineering, University of Castilla-La Mancha, Ronda de Calatrava 7, 13071 Ciudad Real, Spain; mariaelena.alanon@uclm.es; 4 Departamento de Ingeniería Agroforestal, Escuela Politécnica Superior, Universidad de Santiago de Compostela, Campus Universitario s/n, 27002 Lugo, Spain; ignacio.diazmaroto@usc.es

**Keywords:** shelf life, storage, cork, wine, phenolic compounds, volatile compounds, sensorial

## Abstract

The effects of different storage conditions, light exposure, temperature and different commercially available cork stoppers on the phenolic, volatile and sensorial profile of Verdejo wines were studied. Two natural corks of different visual quality and a microgranulated cork stopper were investigated over one year at two different storage conditions. One simulating light exposure and temperature in retail outlets and the other simulating optimal cellar conditions (darkness and 12 °C). The wines stored under commercial conditions showed greater losses of total and free SO_2_ and higher levels of brown-yellowish tones, related to the oxidation of flavan-3-ols. Although these wines underwent a decrease in the total content of stilbenes, a significant increase in *trans*-piceid was observed. In addition, these wines suffered important changes in their volatile and sensory profile. Volatile compounds with fruity and floral aromas decreased significantly, while volatile compounds related to aged-type characters, as linalool oxides, vitispirane, TDN or furan derivatives increased. Wines stored in darkness at 12 °C underwent minor changes and their sensory profiles were similar to wine before bottling. The high-quality natural corks and microgranulated corks better preserved the quality of the white wines from a sensory point of view. These results showed that temperature and light exposure conditions (diffuse white LEDs and 24 ± 2 °C) in retail outlets considerably decrease the quality of bottled white wines and, consequently, their shelf life, due to the premature development of aged-type characters.

## 1. Introduction

Dry white wines are normally consumed within a short period of time after bottling. Their quality at the time of consumption will be closely related to their chemical composition and sensory characteristics before bottling, although storage conditions such as temperature, light exposure or oxygen transmitted through cork stoppers will determine the evolution of their initial quality [1].

Exposure to oxygen, light or high temperatures of white wines during storage has a negative effect on their quality. Inadequate storage will cause the development of oxidized characters, protein instability, and an alteration of their aroma related to the loss of volatile compounds responsible for the fruity character and other fermentation compounds [2,3,4]. White wines are sensitive to oxidation, and there is no way to completely block wine from exposure to oxygen. Furthermore, a complete absence of oxygen after bottling could lead to the development of undesirable reduction aromas [5,6].

There are several studies about the effect of the oxygen on the chemical and sensorial composition of dry white wines during aging. They mainly focused on the influence of oxygen concentration, together with other factors as temperature, pH or SO_2_ concentration, on the development of undesirable compounds [7,8]. Some of these studies tried to reproduce the degradation of the aroma of dry white wine with accelerated oxidative aging [2] while other researchers monitored the effect of a controlled oxidation on certain chemical and sensory parameters [9].

Oxygen exposure considerably decreases the quality of white wines. However, wine producers tend to bottle their white wines under an inert atmosphere, such as one consisting of nitrogen, to enhance protection. Different studies have suggested that the deterioration of white wines in the bottle is due more to the low diffusion of oxygen through the cork and to the physiological release of oxygen from cork pores during its insertion in the neck at bottling than to the concentration of oxygen present in the wine when bottled [10,11]. For example, wines sealed with synthetic closures developed earlier oxidized characters in aroma and color than wines sealed with natural or agglomerate corks, due to their different degree of porosity and pore size [5,6,10]. While screw-cap-sealed wines better maintained their initial typical appearance and aroma, they showed certain reduction attributes [6,10].

However, during bottle aging, the evolution of the chemical and sensory composition of white wines does not only depend on the oxygen concentration. Other parameters such as concentration of transition metal ions, temperature and light exposure are essential to achieve the preservation of wine quality and extend its useful life. The storage temperature has an important influence on the kinetics of the chemical reactions that occur during aging [12]. As the temperature increases, the molecules move faster and increase the possibility of colliding more frequently, increasing the reaction rate. Red wines stored at 25 °C for 24 months underwent significant changes in their chemical composition and sensory attributes, with high organoleptic perceptions of dried fruit aromas [13]. Higher temperatures induced the largest sensory and chemical changes in Chardonnay wines storage during 3 months with different packaging types [14]. Sauvignon Blanc and Chenin Blanc wines stored at 25 °C for 9 months showed greater values in parameters related to browning [15].

A wine can experience high temperatures in different instances of its life, for example, during aging, transport, storage or sale. All these points are critical and sometimes combine high temperatures with exposure to light, as occurs in retail outlets. The photogeneration of sulfur volatile compounds in Champagne wines exposed to light of wavelengths below 450 nm was previously observed [16]. Ultraviolet light and, to a lesser extent, low-wavelength visible light can contribute to pigment enhancement of white wines [17]. Clark et al. [18] demonstrated the potential for iron (III) tartrate to act as a photoactivator in visible (below 520 nm) light-induced oxidative degradation of white wine. Exposure to light could be reduced by choosing darker colored bottles; however, today, white wines are still mostly bottled in transparent bottles due to consumer demands. Días et al. [17] studied the effect of bottle color on pigment enhancement in a Chardonnay white wine and observed that none of the bottle glass color totally prevented the light-induced color development of the wine.

In the literature, there are several studies on the effect of different storage conditions on chemical and sensory parameters of wine. Unfortunately, most of these studies focus on specific compounds and few carry out an extensive study of all the parameters that can influence in the quality of a wine. In this work, a complete study of the evolution of aroma attributes, color parameters and volatile and phenolic profiles of bottled white wines during their storage was realized. This research was designed to compare the effect of light exposure, temperature and type of cork on the quality of a bottled Verdejo white wine for one year. Two storage conditions were chosen: one simulating light exposure and temperature in retail outlets (cyclic light/dark 12 h/12 h to white LEDs and 24 ± 2 °C) and another simulating optimal cellar conditions (darkness and 12 °C). All the wines were bottled in clear glass bottles with three different corks: two natural corks of different visual quality grades, super and third, and a microgranulated cork.

## 2. Results and Discussion

### 2.1. Conventional Enological Parameters

Table 1 shows the conventional enological parameters of the Verdejo wine just before bottling. All the values obtained are typical for that kind of wine and generally remained stable during bottle aging, although some variations were detected in the acetic acid and free and total SO_2_ contents. The concentration of the acetic acid varied from 0.08 g/L in the initial wine to 0.21 g/L in the sample storage with the lowest quality natural corks (THR) under commercial conditions (light and room temperature). No differences were found between wines bottled with high-quality natural corks (SUP) and microgranulated corks (MGR).

**Figure 1:**
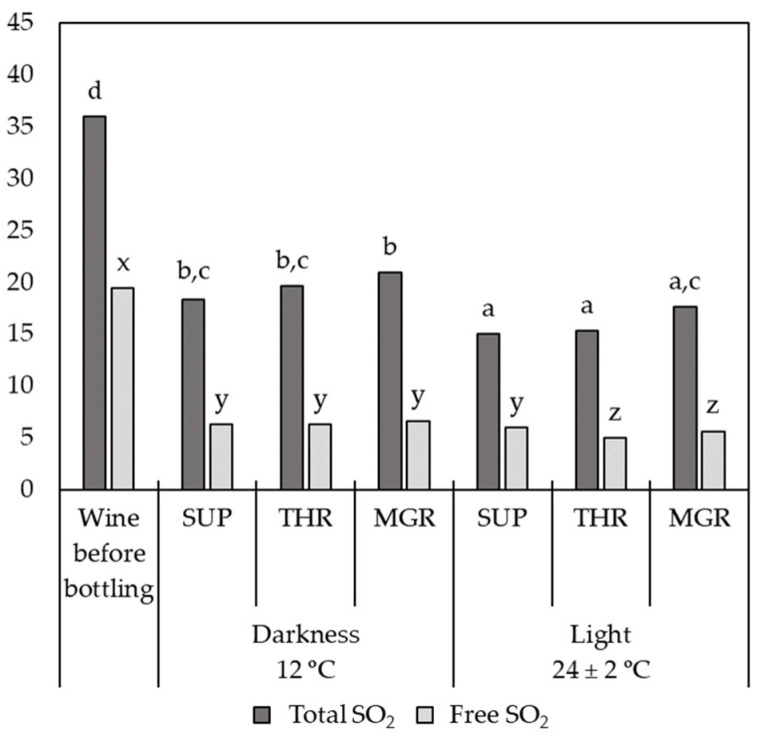
Total and free SO_2_ levels (mg/L) in Verdejo wines before bottling and those storage for one year under the different conditions tested. SUP: super quality natural corks; THR: third quality natural corks; MGR: microgranulated corks. Different letters (a,b,c,d or x,y,z) in the same variable mean significant differences (α = 0.05) according to the test of Student–Newman–Keuls.

The effect of the bottle aging conditions on the levels of free and total SO_2_ was clearly observed. Figure 1 shows the initial and final content of free and total SO_2_ in the samples grouped according to their storage conditions. Both parameters significantly decreased in all wines compared to the wine before bottling. This decrease was greater in wines stored simulating commercial conditions (light exposure and room temperature), with average losses of 55.6% for total SO_2_ and 71.3% for free SO_2_, while SO_2_ lost in wines stored at 12 °C and darkness were 42.3% and 67.2% for total and free SO_2_, respectively. Regarding the different types of corks used, no statistically significant differences were found. The level of free SO_2_ fell below 10 mg/L after one year in all wines, regardless of storage conditions, which implies a lack of protection for these white wines [19], and, consequently, a difficulty to continue their bottle aging.

### 2.2. Color Related Parameters and Phenolic Compounds

Table 2 shows the flavan-3-ols (mg/L), stilbenes (µg/L) and color-related parameters in wines before bottling and those bottle aging for one year under the different conditions studied. Regarding chromatic characteristics, all bottled wines had high level of lightness (L*), and no significant differences were found respect to the wine before bottling. Similar results were found in bottled Chardonnay wines stored at 15 °C with and without light exposure [20]. However, important changes were observed in other parameters. During bottle aging, an evolution in the a*, b*, color intensity (CI) and chroma (C*_ab_) values was obtained. The b* values displayed an important increase in all wines, while the values of a* only slightly rose in wines stored in the dark. All bottle wines presented higher color intensity, regardless of storage conditions, while the values of C*_ab_ were higher in wines stored under commercial conditions. These results agree with those obtained by Hernanz et al. [21], who observed that Zalema and Colombard white wines stored at lower temperatures were less greenish and yellowish and exhibited less colorfulness than those stored at higher temperatures.

Optical density at 420 nm (A420) is a commonly used measure to determine the color change produced in white wines due to their oxidation, and white wines undergo a significant increase in storage in the presence of oxygen; however, in its absence, the increase is quite minor [9]. The bottle-aged wines presented higher values of A420 than wine before bottling; that indicated a change in the color of the wines from pale yellow to a slight yellowish-brown tone, mainly in those stored under commercial conditions. This, together with their low free SO_2_ content, implies a significant risk of the color evolution of these wines towards more accentuated brown-yellowish tones [19,22].

The changes of color observed may be related to modifications in the content of the phenolic compounds. Flavan-3-ols, especially (+)-catechin, (−)-epicatechin and procyanidins, are highly correlated with the browning of white wines [23]. After 12 months of storage in a bottle, a significant loss of flavan-3-ols was observed in all the samples. Important losses of flavan-3-ols during the storage of white wines of different grape varieties have been described by other authors [20,21,24]. In this work, the loss was notably greater in wines stored at room temperature and light exposure, and no differences were observed between the different types of corks used. However, small differences were found in the wines stored in the dark, specifically in the content of (−)-epicatechin, (+)-gallocatechin and an unknown procyanidin, whose concentration was slightly higher in wines sealed with high-quality natural and microgranulated corks, compared with those sealed with low-quality natural corks.

The browning of wines is related to the formation of pigments as a result of oxidative processes. Therefore, the lower decrease in flavan-3-ols observed in wines stored in the dark at 12 °C could be due to less oxidation of monomeric flavan-3-ols to quinones, which polymerize giving rise to macromolecules with brown-yellowish tones [25]. Furthermore, another browning pathway in white wines may be the production of xanthylium cation pigments, formed by the reaction between flavan-3-ols and glyoxylic acid [18,26]. Glyoxylic acid is a degradation product of tartaric acid, whose concentration increases during the photo-oxidation of white wines [18]. These authors demonstrated the potential for iron (III) tartrate to act as a photo-activator in light-induced oxidative degradation of white wines, placing the critical spectral region for this photoactivation below 520 nm. An important decrease in tartaric acid occurred in wines stored with light exposure (data not shown). Therefore, that oxidative pathway could justify the more significant loss of flavan-3-ols observed in wines stored simulating the conditions of retail outlets. These results are in good agreement with those of Dias et al. [17], who studied the role of light and temperature on pigment enhancement in Chardonnay white wine with high flavan-3-ol content. These authors concluded that low wavelength visible light contributes to pigment production; however, wines stored in darkness require temperatures above 50 °C for pigment formation become apparent. 

Four stilbenes were identified in Verdejo wines: *trans*-resveratrol, its glucoside *trans*-piceid and their corresponding *cis* isomers. *trans*-Resveratrol was the main stilbene identified in the wine before bottling, followed by *cis-*piceid, while their isomers appeared in much lower concentrations. Few changes were observed during storage of the wines in darkness at 12 °C. However, stilbenes underwent important modifications in the wines stored under commercial conditions, resulting in a significant decrease of total stilbene content, mainly due to the decrease of *trans* and *cis*-resveratrol and *cis*-piceid. This decrease was greater in the bottled wines sealed with low-quality natural corks, obtaining similar results in the wines sealed with high-quality natural and microgranulated corks. Kolouchová et al. [27] observed a significant decrease in *cis*-resveratrol in wines stored at room temperature in six weeks, while its concentration remained stable in wines stored at 4 °C. Souza et al. [28] observed a decrease in stilbenes in red wines stored at temperatures between 24 and 30 °C for one year, regardless of the color of the bottle used.

*trans*-Piceid has been identified as the major stilbene in different commercial white wines, with concentrations higher than its *cis* isomer, although the storage conditions of these wines are not specified [29,30]. In our study, higher concentrations of *trans*-piceid than *cis*-piceid were observed in wines stored with light at room temperature, whereas the concentration of *cis*-piceid was higher than *trans*-piceid in the wine before bottling and the wine stored in the dark at 12 °C. These results agree with those obtained in previous studies by our research group, where *cis*-piceid was identified in higher concentrations than its *trans* isomer in freshly made white wines [31]. These results show the potential usefulness of both compounds as markers of the storage conditions of white wines.

*trans*-Piceid content increased significantly in bottle-aged wines at commercial conditions, while its *cis* isomer decreased. It is known that *trans*-resveratrol in solution and exposure to light converts to its *cis* isomer. However, the conversion of *trans*-piceid to its *cis* isomer is deterred by the steric hindrance conferred by the sugar moiety [32]. In the solid state, with or without light, *cis*-resveratrol quickly returns to the *trans* form, the thermodynamically more stable isomer [33]. Therefore, the increase in *trans*-piceid and the decrease in its *cis* isomer observed in bottle-aged wines under light and room temperature could be due to the search for stability of the *cis* isomer under improper storage conditions.

### 2.3. Volatile Compounds and Sensorial Profile

In general, several studies have evaluated the effect of different storage conditions on the volatile composition of different white wines. However, most focus on specific groups of compounds and do not carry out a complete study of the volatile profile of wines. Table 3 shows the major and minor volatile compounds identified in the wines before bottling and after one year of bottle aging in the different storage conditions tested. Within the group of major volatiles, significant losses were observed in the content of methanol, diacetyl, ethyl butyrate, isobutanol, isoamyl acetate, isoamyl alcohol and acetoin in bottle-aged wines. These losses were higher in wines stored with light at room temperature. In these wines, there was a significant decrease in ethyl acetate, which was not observed in wines stored in darkness at 12 °C. However, not only did volatile losses occur, but some compounds did not undergo changes in their concentration, such as the higher alcohols propanol and 1-butanol and acetaldehyde, while the content of ethyl lactate increased significantly in bottle-aged wines. Similar results were found in commercial white wines stored without temperature control compared to those stored at 5 °C [3] and in Chardonnay wines stored at 15 °C [20]. In this last study, the authors did not find any difference between the wines stored with or without light exposure. 

A total of 61 minor volatile compounds belonging to different chemical families (esters, C6-alcohols, benzenic compounds, terpenes, C13-norisoprenoids, lactones, furan and pyran compounds, acids and sulfur compounds) were selected to study the effect of bottle aging in Verdejo wines. Ethyl esters and acetates were the largest group. These compounds are formed during alcoholic fermentation, and most impart pleasant fruity notes to white wines, such as Verdejo wines, in which they are usually found above their olfactory perception thresholds [34]. Esters are susceptible to hydrolysis–esterification reactions, which are mainly influenced by the storage temperature, which justifies the changes observed in their concentration during the storage of the wines [12]. There was a significant decrease in short- and medium-chain fatty acid ethyl esters and acetates in all bottled wines, since they are hydrolyzed until reaching equilibrium conditions [3,12]. However, other esters showed higher concentrations after one year of storage, especially ethyl succinate and ethyl glutarate, due to the higher concentration of their corresponding acids in wines. This increase was significantly higher in wines stored under commercial conditions. The same behavior has been previously observed by other authors, highlighting the importance of temperature during the storage of white wines [3,20,35].

Within the group of benzene compounds, the main differences were found in 2-phenylethyl acetate, whose concentration decreased in all bottled wines, mainly, like other acetates, in wines stored at higher temperatures.

Several terpenes were identified in Verdejo wines, which underwent significant changes in their concentration with storage. Within the monoterpenic alcohols, all samples presented lower concentration of linalool and citronellol, and higher concentration of α-terpineol, than wine before bottling. These changes were especially significative in the wines stored simulating the conditions in retail outlets. In addition to α-terpineol, other terpenes whose concentration was higher in bottle-aged wines were linalool oxides, although this increase only occurred in wines stored with light at room temperature. During the conservation of wine in the bottle, both acid hydrolysis of terpenic glycosides can occur, which would enrich the wine in monoterpenols, as well as molecular transformations of monoterpenols through different reactions of isomerization, cyclization, hydration, dehydration or oxidation, giving rise to odorous terpenes as linalool oxides, hotrienol or α-terpineol [36]. Other authors have observed similar results during bottle aging of white wines, with a greater degradation of terpenes at high temperatures and light exposure [14,20,35].

Together terpenes, C13-norisoprenoids play an important role in the aroma of young Verdejo wines [34]. This group consisted of compounds with floral and fruity notes, such as β-damascenone (floral and peach), α-ionone (violets, fruity) and 3-oxo-α-ionol (floral, tobacco), and by two compounds with aged-like aromas, vitispirane and 1,1,6-trimethyl-1,2-dihydronaphthalene (TDN). There was a significant decrease of β-damascenone and 3-oxo-α-ionol in bottle-aged wines, regardless of storage conditions, whereas vitispirane and TDN appeared in higher concentration in wines stored at room temperature and light exposure. C13-norisoprenoids are subjected to acid-catalyzed reactions analogous to those observed in terpenes and can increase or decrease during storage of bottled wines [36]. TDN is normally formed in wines stored in the bottle for long periods of time; however, the storage of wines for a year simulating commercial conditions strongly stimulated the production of this compound and vitispirane. The premature increase of these compounds has been previously observed during the storage of white wines, mainly at high temperatures and regardless of the light exposure [20,37].

A significant increase in furan derivatives was observed in bottle-aged wines stored at room temperature and light exposure, such as furfural, furanyl ethanone, ethyl-2-furoate, 5-ethoxymethylfurfural, furaneol and 5-hydroxymethylfurfural. Furfural can be formed by oxidation of ascorbic acid, although most of these compounds increase due to the degradation of residual sugars, so they do not usually participate in the aroma of bottle-aged wines since they do not reach concentrations higher than their olfactory perception thresholds [36].

According to other studies, bottle-aged wines did not show changes in the main volatile acids and C6-alcohols [3,15], whereas some changes were observed in the group of lactones. The concentration of γ-caprolactone increased in all bottled wines, while the content of γ-nonalactone and γ-decalatone was higher only in wines stored at room temperature and light exposure.

Several middle and higher boiling sulfur compounds formed during alcoholic fermentation were identified. These compounds have generally high detection thresholds and do not typically cause defects [38]. Their concentration decreased during bottle aging of the wines, especially in those stored in darkness at 12 °C.

**Figure 2:**
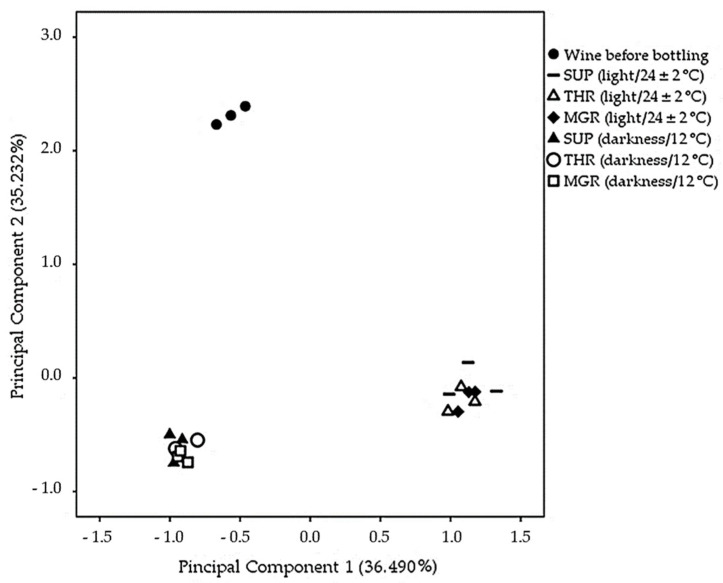
Plot of wines distributed in the space defining by principal components PC1 and PC2 regarding significant minor volatile compounds. SUP: super quality natural corks; THR: third quality natural corks; MGR: microgranulated corks.

In order to identify the variables that most contributed to the differentiation of the wines, the principal component analysis (PCA) was applied to the minor volatile components shown in Table 3. The first two principal components (PC) explained 71.7% of the variance. The distribution of the samples in the space formed by these two components is shown in Figure 2. PC 1 (36.5%) separated the bottle-aged wines at room temperature and light exposure from the rest. The compounds positively correlated with this component were mainly four terpenes, linalool oxides, α-terpineol and hotrienol, two C13-norisoprenoids, vitispirane and TDN, and several furan and pyran compounds, whose concentration increased in the wines stored under commercial conditions. Linalool appeared negatively correlated with principal component 1 due to the significant losses observed in these wines.

PC 2 explained 35.2% of the variance between samples and separated wine before bottling from bottle-aged wines, regardless of their storage conditions. This component was positively correlated with ethyl esters, acetates, citronellol, and two C13-norisoprenoids, β-damascenone and 3-oxo-α-ionol, whose concentration decreased in all bottle-aged wines.

Regarding the different cork stoppers tested, the PCA could not establish groups. However, small differences were found in specific volatile compounds, such as ethyl octanoate, ethyl 2-hydroxy-4-methylpentanoate and ethyl decanoate, whose losses were higher in bottled wines sealed with low-quality natural corks.

**Figure 3:**
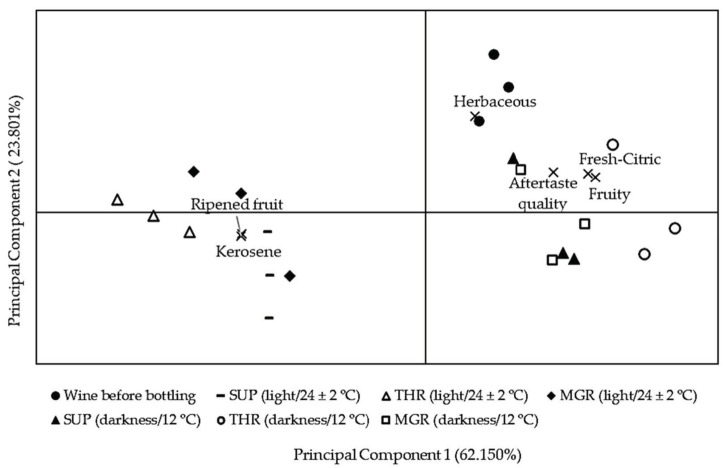
Distribution in the space of wines before bottling and those storage for one year under the different conditions tested, joint with their sensorial attributes (x). SUP: super quality natural corks; THR: third quality natural corks; MGR: microgranulated corks.

Wines showed changes in their aroma profile during bottle aging. Figure 3 presents the principal component biplot, illustrating the simultaneous projection of wines and their sensorial descriptors. The first two principal components explained 86.0% of the total variance. PC1 and PC2 accounted for 62.2% and 23.8% of the variance between samples, respectively. As in the PCA of the volatile compounds, wines were grouped into three groups, wine before bottling, wines stored simulating light exposure and temperature in retail outlets and wines simulating optimal cellar conditions (darkness and 12 °C).

Verdejo wines before bottling were described as fresh with intense herbaceous, citrus and fruity notes. Bottle-aged wines stored in darkness at 12 °C retained these sensory attributes, although their intensity decreased slightly during storage, mainly in wines sealed with low-quality natural corks. This decrease was especially important in wines stored at room temperature and light exposure, which also showed differences between wines sealed with high-quality natural and microgranulated corks and wines sealed with low-quality natural corks. The decrease in ethyl esters, acetates, monoterpenols as linalool and citronellol, and C13-norisoprenoids as β-damascenone and 3-oxo-α-ionol can contribute to the decrease in the intensity of the characteristic sensory notes of Verdejo wines. These results are in good agreement with those of Pérez-Coello et al. [3], who suggested that storage of young white wines at 5 °C increases their shelf-life, since produces less chemical and sensory changes over time than storage at room temperature. Recamales et al. [39] showed less loss of fruitiness in Zalema white wines after 12 months of storage in bottle at low temperature. In this study, the temperature had the greatest impact on the volatile and sensorial profile of wines, compared with other factors as time storage or light exposure. These findings have recently been supported by Ling et al. [40], who reported a loss of fresh and fruity characters in Chardonnay white wines stored at 16 °C over time.

Two new descriptors were detected by the judges in wines stored at room temperature and light exposure, ripened fruit and kerosene, which were evaluated with greater intensity in wines sealed with the lowest quality natural corks. TDN has a characteristic kerosene aroma, with a perception threshold close to 20 µg/L [41], a value exceeded by all stored wines simulating light exposure and temperature in retail outlets. Silva Ferreira et al. [37] concluded that the combined effect of pH and temperature was responsible for the formation of TDN, vitispirane and linalool oxides, and attributed to these compounds off-flavors such as farm feed and rotten food; however, these aromas were not detected in our wines.

Finally, the judges evaluated the aftertaste quality of the wines and gave a similar score to the wines before bottling and those maintained in darkness at 12 °C. The wines stored at room temperature and light exposure presented a lower aftertaste quality. Regarding the different types of cork stoppers studied, no differences were found between wines sealed with high-quality natural and microgranulated corks, while wines sealed with low-quality natural corks received the lowest scores, in both storage conditions. Moreira et al. [42] reported similar sensorial profiles in wines sealed with average-quality natural and technical corks.

## 3. Materials and Methods

### 3.1. Cork Samples

Three commercially available cork stoppers from *Quercus suber* L. oak trees were provided by different manufacturers. Two of them were natural corks of different qualities (visual level), super (SUP) and third (THR), while the other one was a microgranulated cork stopper (MGR) made with natural cork byproducts. The dimensions of the corks were 44 × 24 mm. Super and microgranulated corks (SUP and MGR) were made with cork planks from South Spain whereas the low-quality natural corks (THR) were fabricated with planks from Portugal.

The moisture content of the samples was determined in duplicate according to the UNE 56921 standard [43]. Two grams (2 g) of homogenous samples were dried using a (Selecta, Barcelona, Spain) laboratory oven at 100 °C until there was no difference in weight between two consecutive measurements. The humidity values were 3.32 ± 0.25, 3.65 ± 0.15 and 3.14 ± 0.21 for SUP, THR and MGR, respectively.

### 3.2. Storage Conditions

A commercial Verdejo (*Vitis vinifiera*) white wine from 2018 vintage produced in Ciudad Real (Spain) was bottled with three different cork stoppers described above and bottle-aged under different storage conditions for one year. Bottles were supplied by Vinícola de Castilla (Manzanares, Ciudad Real, Spain). The bottles used were transparent and 750 mL of volume. The wine was manually filled into bottles at 50 ± 2 mm from the top of the bottle. Prior to the insertion of the cork stoppers, bottles were sealed under a flush of nitrogen. Bottled wines were then divided into two batches. One batch was stored under optimal cellar conditions, darkness and 12 °C, and the other batch was stored simulating commercial conditions in retail outlets, low visible light exposure during 12 h at room temperature (24 ± 2 °C). White light emitting diodes (white LEDs) were used for the diffuse illumination of the wines. The color temperature was 6000 k and the wavelength was 483 nm. All the storage conditions were tested in triplicate.

### 3.3. Conventional Analysis and Color Parameters

Alcoholic degree, total acidity, pH, acetic acid, free and total SO_2_, pH, glucose and fructose, organic acids (malic, lactic, citric and succinic), glycerol, meso- and levo-butanediol were determined by official analytical methods established in the International Organization of Vine and Wine [44].

Color parameters were obtained according to the OIV method for the determination of chromatic characteristics in the CIELAB space. The measurements were made in an Agilent 8453 diode array spectrophotometer (Agilent Technologies, Santa Clara, CA, USA) under the following conditions: transmittance between 770 and 380 nm at 5 nm intervals, 1 mm cuvettes, D65 illuminant and a 10° reference pattern observer. Results expressed were referred to 1 cm optical length [44].

### 3.4. Analysis of Phenolic Compounds

The flavan-3-ols and stilbenes were analyzed in an HPLC Agilent 1200 series system equipped with DAD (Agilent, Waldbronn, Germany), and coupled to an AB Sciex 3200 TRAP (Applied Biosystems, Foster City, CA, USA) with triple quadrupole, turbo spray ionization (electrospray assisted by a thermonebulization, Agilent, Waldbronn, Germany) and a mass spectroscopy system (ESI-MS/MS, Agilent, Waldbronn, Germany). They were previously isolated from wine by SPE on C18 cartridges (Waters Sep-Pak Plus, 820 mg of adsorbent, Saint-Quentin En Yvelines, France) and then separated in an Ascentis-C18 column (4.6 × 150 mm; 2.7 μm particle; Supelco, Germany), thermostated at 16 °C and with a flow rate of 0.3 mL/min [45].

Pure standards were used for identification and quantification (calibration curves): (+)-catechin, (−)-epicatechin, *trans*-resveratrol and *trans*-piceid from Sigma Aldrich (Tres Cantos, Madrid, Spain); (+)-gallocatechin and (−)-epigallocatechin from Phytolab (Vestenbergsgreuth, Germany); procyanidins B1 and B2 from Extrasynthese (Genay, France). *cis*-Resveratrol and *cis*-piceid were obtained by UV irradiation (366 nm during 5 min in quartz vials) of a 25% MeOH solution of their *trans* isomers.

### 3.5. Analysis of Volatile Compounds

Major volatile compounds were analyzed by GC-MS (Focus-ISQ, Thermo Scientific, Milan, Italy). 100 μL of wine was mixed with 100 μL of internal standard (4-methyl-2-pentanol, 60.35 mg/L) and 1 mL Milli-Q water. Then, 1 µL of the mixture was injected in split mode into a BP-21 column (60 m × 0.32 mm × 0.25 µm) FFAP phase (polietilenglicol modified with nitrotereftalic acid, TPA). Helium was used as carrier gas at a constant flow of 1.2 mL/min. The injector temperature was 195 °C and oven temperature was set at 32 °C during 2 min, ramped at 5 °C/min to 120 °C, then increased at 75 °C/min to 190 °C and maintained during 18 min. MS operated in the electron impact mode at 70 eV and the ion source temperature was 250 °C. Identification was carried out by comparison with commercial standards (Sigma-Aldrich Chemie GmbH, Steinheim, Germany), which were used to do the calibration curves for the quantification of the major volatile components.

Minor volatile compounds of wines were extracted by solid phase extraction (SPE). 100 mL of wine, together with 40 µL of 4-nonanol (1 g/L) as internal standard, was fractioned in 500 mg of previously conditioned styrene divinyl benzene cartridges (Lichrolut EN Merck, KGaA, Darmstadt, Germany). Nonvolatile hydrophilic compounds were washed out of the cartridges with 50 mL of Milli Q water, and minor volatiles were eluted with 10 mL of dichloromethane. Extracts were concentrated to 200 µL under stream of nitrogen and stored at −20 °C prior to their GC-MS analysis.

One microliter (1 µL) of the extracts was injected in splitless mode (0.4 min) in a 6890 N Agilent gas chromatograph coupled to a 5973 N Agilent Mass Detector, and fitted with a DB-WAX ultra-inert column, 60 m × 0.25 mm i.d.; 0.25 μm film thickness (Agilent, Waldbronn, Germany). The injector temperature was 250 °C and the oven temperature was programmed as follows: 70 °C (5 min) first ramped at 1 °C/min to 95 °C (10 min) and then at 2 °C/min until 210 °C, that was held for 40 min. The carrier gas was helium at a flow of 1 mL/min. MS operated in the electron impact (EI) ionization mode at 70 eV, the ion source temperature was 230 °C, and spectra were recorded in the SCAN mode (45 to 550 a.m.u.).

Identification of the volatile components was performed comparing with authentic standards from Sigma-Aldrich (Steinheim, Germany). The tentative identification of compounds for which it was not possible to find reference volatiles was carried out by comparison of their mass spectra with spectral data from the Wiley G 1035 A, NBS75K and NIST14 libraries and by the linear retention index (LRI) comparison. Response factor of each volatile compound was calculated by injection of commercial standards. For compounds that commercial standards were not available, the response factors of compounds with similar chemical structures were used (ethyl 2-hydroxy-3-methylbutyrate, methyl 2-hydroxy-4-methylpentanoate, ethyl 2-hydroxy-4-methylpentanoate and ethyl 4-hydroxybutyrate were quantified as ethyl 3-hydroxybutyrate; hotrienol and hydroxylinalool as linalool; vistispirane and TDN as β-damascenone; 3-oxo-α-ionol as α-ionone; methyl propanoic acid as hexanoic acid; 2-methyl-dihydro-thiophen-3-one, ethyl 3-methylthiopropanoate, and 3-methylthiopropanoic acid as methionol).

### 3.6. Sensorial Analysis

Descriptive sensory analysis was used to evaluate wine organoleptic characteristics. Wine evaluations took place in standard sensory analysis chambers (ISO 8589:2007) using wine testing glasses (ISO 3591:1997). Eight judges evaluated the wines according to the ISO 11035:1994 where the identification and selection of descriptors for establishing a sensory profile by a multidimensional approach is defined. In previous sessions, judges generated sensory terms individually and a tasting sheet with six attributes was created by consensus, which described the wine sensorial profile. Then, judges sniffed and tasted the wines by duplicate in two sessions, using a 10 cm unstructured scale to rate the intensity of each attribute. The left extreme of the scale indicated a null intensity of the descriptor and the right extreme indicated the maximum values.

### 3.7. Statistical Analysis

Statistical analysis was carried out using the IBM SPSS software, version 24.0 (IBM, Madrid, Spain) for windows statistical package. The Student–Newman–Keuls test was applied to the chemical data to identify statistical differences among different storage conditions. Minor volatile compound data and sensorial data were subjected separately to principal component analysis (PCA) to highpoint the main contributors to the variance among wines.

## Figures and Tables

**Table 1:** Enological conventional parameters of the Verdejo wine before bottling.

Conventional Parameter	Value (*n* = 3)
alcoholic degree (% *v*/*v*)	11.9 ± 0.0
total acidity (g/L)	5.03 ± 0.05
pH	3.27 ± 0.00
total SO_2_ (mg/L)	36.0 ± 1.7
free SO_2_ (mg/L)	19.5 ± 0.5
glucose and fructose (g/L)	1.75 ± 0.01
acetic acid (g/L)	0.08 ± 0.01
malic acid (g/L)	1.60 ± 0.00
citric acid (g/L)	0.33 ± 0.02
tartaric acid (g/L)	2.14 ± 0.07
succinic acid (g/L)	0.60 ± 0.02
lactic acid (g/L)	0.02 ± 0.01
glycerol (g/L)	5.69 ± 0.20
meso-butanediol (g/L)	0.10 ± 0.01
levo-butanediol (g/L)	0.23 ± 0.01

**Table 2:** Flavan-3-ols (mg/L), stilbenes (µg/L) and color-related parameters in wines before bottling and storage for one year under the different conditions tested. SUP: super quality natural corks; THR: third quality natural corks; MGR: microgranulated corks.

	Wine before Bottling	Light Exposure/24 ± 2 °C			Darkness/12 °C		
		SUP	THR	MGR	SUP	THR	MGR
**Flavan-3-ols (mg/L)**							
(+)-catechin	3.96 ^c^ ± 0.24	1.26 ^a^ ± 0.11	1.14 ^a^ ± 0.12	1.26 ^a^ ± 0.07	2.65 ^b^ ± 0.08	2.41 ^b^ ± 0.11	2.66 ^b^ ± 0.16
(−)-epicatechin	2.64 ^d^ ± 0.02	0.43 ^a^ ± 0.03	0.40 ^a^ ± 0.05	0.42 ^a^ ± 0.04	1.40 ^c^ ± 0.03	1.27 ^b^ ± 0.07	1.40 ^c^ ± 0.06
(+)-gallocatechin	0.87 ^d^ ± 0.01	0.40 ^a^ ± 0.00	0.36 ^a^ ± 0.03	0.37 ^a^ ± 0.03	0.68 ^c^ ± 0.04	0.57 ^b^ ± 0.03	0.64 ^c^ ± 0.02
(−)-epigallocatechin	0.16 ^c^ ± 0.00	0.05 ^a^ ± 0.02	0.04 ^a^ ± 0.01	0.05 ^a^ ± 0.01	0.11 ^b^ ± 0.01	0.10 ^b^ ± 0.01	0.12 ^b^ ± 0.02
procyanidin B1	0.70 ^c^ ± 0.03	0.02 ^a^ ± 0.00	0.02 ^a^ ± 0.00	0.03 ^a^ ± 0.00	0.41 ^b^ ± 0.03	0.37 ^b^ ± 0.02	0.40 ^b^ ± 0.05
procyanidin B2	0.29 ^c^ ± 0.01	0.02 ^a^ ± 0.00	0.01 ^a^ ± 0.00	0.02 ^a^ ± 0.00	0.19 ^b^ ± 0.02	0.18 ^b^ ± 0.02	0.19 ^b^ ± 0.02
procyanidin unknown	0.20 ^d^ ± 0.00	0.01 ^a^ ± 0.00	0.01 ^a^ ± 0.01	0.01 ^a^ ± 0.00	0.08 ^c^ ± 0.01	0.07 ^b^ ± 0.01	0.07 ^b^ ± 0.01
monomers glycosides	3.24 ^c^ ± 0.00	2.36 ^a^ ± 0.14	2.23 ^a^ ± 0.11	2.28 ^a^ ± 0.04	2.76 ^b^ ± 0.10	2.67 ^b^ ± 0.08	2.83 ^b^ ± 0.16
**Stilbenes (µg/L)**							
*trans*-piceid	54.0 ^a^ ± 0.3	391.9 ^b^ ± 27.1	435.2 ^c^ ± 23.6	393.8 ^b^ ± 21.3	61.1 ^a^ ± 2.5	62.5 ^a^ ± 0.8	67.5 ^a^ ± 3.5
*cis*-piceid	512.8 ^d^ ± 3.6	153.5 ^b^ ± 4.2	85.4 ^a^ ± 5.2	135.2 ^b^ ± 8.9	526.1 ^d^ ± 10.8	483.8 ^c^ ± 14.8	498.6 ^c,d^ ± 19.2
*trans*-resveratrol	614.0 ^d^ ± 9.5	180.2 ^b^ ± 9.2	97.6 ^a^ ± 17.2	182.6 ^b^ ± 6.6	629.7 ^d^ ± 17.7	554.3 ^c^ ± 22.3	608.1 ^d^ ± 22.1
*cis*-resveratrol	64.9 ^c^ ± 2.7	24.7 ^b^ ± 2.5	13.3 ^a^ ± 1.7	17.3 ^a,b^ ± 2.7	95.2 ^d^ ± 5.9	72.0 ^c^ ± 6.9	91.6 ^d^ ± 5.0
**Color parameters**							
L*	96.80 ^a,b,c^ ± 0.12	97.03 ^b,c^ ± 0.06	97.40 ^c^ ± 0.13	97.23 ^c^ ± 0.12	96.56 ^a,b^ ± 0.08	96.45 ^a,b^ ± 0.31	96.30 ^a,b^ ± 0.58
a*	−0.63 ^a^ ± 0.04	−0.66 ^a^ ± 0.07	−0.60 ^a^ ± 0.08	−0.72 ^a^ ± 0.04	−0.42 ^b^ ± 0.02	−0.41 ^b^ ± 0.06	−0.40 ^b^ ± 0.11
b*	4.35 ^a^ ± 0.30	12.81 ^c^ ± 0.17	12.72 ^c^ ± 0.30	12.69 ^c^ ± 0.20	9.94 ^b^ ± 0.21	10.75 ^c^ ± 0.17	10.45 ^b,c^ ± 0.64
*color intensity (CI)*	0.16 ^a^ ± 0.00	0.24 ^b^ ± 0.00	0.22 ^b^ ± 0.01	0.23 ^b^ ± 0.00	0.22 ^b^ ± 0.01	0.23 ^b^ ± 0.01	0.23 ^b^ ± 0.03
C*_ab_	2.45 ^a^ ± 0.11	4.09 ^c^ ± 0.05	4.34 ^c^ ± 0.12	4.23 ^c^ ± 0.13	3.20 ^b^ ± 0.02	3.30 ^b^ ± 0.17	3.16 ^b^ ± 0.24
*A _420 nm (a_._u_._)_*	0.10 ^a^ ± 0.00	0.18 ^c^ ± 0.00	0.18 ^c^ ± 0.00	0.18 ^c^ ± 0.00	0.15 ^b^ ± 0.00	0.16 ^b^ ± 0.01	0.16 ^b^ ± 0.01

^a,b,c,d^ Different letters in the same row mean significant differences (α = 0.05) according to the test of Student–Newman–Keuls.

**Table 3:** Major (mg/L) and minor (µg/L) volatile compounds in wines before bottling and storage for one year under the different conditions tested. SUP: super quality natural corks; THR: third quality natural corks; MGR: microgranulated corks.

	Wine Before Bottling	Light Exposure/24 ± 2 °C			Darkness/12 °C		
		SUP	THR	MGR	SUP	THR	MGR
**Major volatile compounds (mg/L)**							
acetaldehyde	38.5 ^c^ ± 0.7	31.2 ^b^ ± 1.8	28.5 ^a^ ± 1.5	27.4 ^a^ ± 2.2	36.0 ^c^ ± 0.2	32.3 ^b^ ± 0.1	33.5 ^b^ ± 2.1
ethyl acetate	75.5 ^d^ ± 0.7	37.1 ^b^ ± 4.1	29.5 ^a^ ± 1.7	30.7 ^a^ ± 2.0	70.3 ^c,d^ ± 2.4	68.0 ^c^ ± 3.6	73.9 ^c,d^ ± 3.4
methanol	104.6 ^d^ ± 3.9	68.5 ^c^ ± 1.7	63.6 ^a,b,c^ ± 2.7	67.3 ^b,c^ ± 3.1	59.0 ^a^ ± 1.4	61.0 ^a,b^ ± 0.1	63.7 ^a,b,c^ ± 4.7
diacetyl	1.4 ^b^ ± 0.30	nd ^a^	nd ^a^	nd ^a^	nd ^a^	nd ^a^	nd ^a^
ethyl butyrate	0.8 ^b^ ± 0.05	nd ^a^	nd ^a^	nd ^a^	nd ^a^	nd ^a^	nd ^a^
propanol	62.5 ^a,b^ ± 0.1	66.4 ^c^ ± 2.3	61.3 ^a^ ± 1.3	62.4 ^a,b^ ± 2.2	63.0 ^a,b^ ± 2.2	68.9 ^c^ ± 0.3	64.6 ^a,b^ ± 2.2
isobutanol	23.9 ^c^ ± 3.2	13.6 ^a,b^ ± 1.8	12.6 ^a,b^ ± 2.3	13.8 ^a,b^ ± 3.3	14.7 ^a,b^ ± 2.0	17.6 ^b^ ± 1.9	10.4 ^a^ ± 1.7
isoamyl acetate	6.4 ^c^ ± 0.4	0.2 ^a^ ± 0.1	0.2 ^a^ ± 0.1	0.1 ^a^ ± 0.0	2.7 ^b^ ± 0.1	2.5 ^b^ ± 0.3	2.5 ^b^ ± 0.2
1-butanol	0.7 ^a^ ± 0.2	0.7 ^a^ ± 0.2	0.6 ^a^ ± 0.1	0.8 ^a^ ± 0.3	0.9 ^a^ ± 0.2	0.5 ^a^ ± 0.1	0.7 ^a^ ± 0.3
isoamyl alcohol	192.3 ^c^ ± 2.0	174.4 ^b^ ± 4.2	169.8 ^a,b^ ± 6.9	172.8 ^b^ ± 4.1	157.3 ^a^ ± 5.1	167.0 ^a,b^ ± 5.1	167.6 ^a,b^ ± 8.9
acetoin	8.9 ^d^ ± 0.1	0.1 ^a^ ± 0.0	0.1 ^a^ ± 0.0	0.1 ^a^ ± 0.0	2.1 ^c^ ± 0.42	1.3 ^b^ ± 0.2	2.4 ^c^ ± 0.2
ethyl lactate	7.5 ^a^ ± 0.4	19.5 ^c^ ± 1.4	20.5 ^c^ ± 1.6	21.1 ^c^ ± 2.0	14.2 ^b^ ± 0.9	16.6 ^b^ ± 1.3	15.3 ^b^ ± 1.2
**Minor volatile compounds (µg/L)**							
**Esters**							
ethyl hexanoate	661.4 ^b^ ± 39.7	546.8 ^a,b^ ± 68.3	551.9 ^a,b^ ± 70.0	576.7 ^a,b^ ± 34.8	591.6 ^a,b^ ± 56.9	595.4 ^a,b^ ± 63.3	503.9 ^a^ ± 38.4
ethyl pyruvate	34.6 ^b^ ± 6.3	31.4 ^a,b^ ± 8.4	26.3 ^a,b^ ± 5.2	23.4 ^a,b^ ± 9.3	19.9 ^a,b^ ± 7.8	20.2 ^a,b^ ± 2.1	16.1 ^a^ ± 2.8
hexyl acetate	257.2 ^d^ ± 6.3	18.0 ^a^ ± 5.1	24.3 ^a^ ± 8.2	28.8 ^a^ ± 7.2	138.7 ^c^ ± 10.4	152.3 ^c^ ± 13.1	112.6 ^b^ ± 18.2
ethyl 2-hydroxy-3-methylbutyrate	2.9 ^a^ ± 0.2	7.2 ^b^ ± 0.5	7.3 ^b^ ± 0.5	7.7 ^b^ ± 0.2	2.8 ^a^ ± 0.2	2.8 ^a^ ± 0.1	2.5 ^a^ ± 0.5
ethyl octanoate	1626.1 ^c^ ± 9.3	560.5 ^b^ ± 89.8	428.8 ^a^ ± 50.5	440.0 ^a^ ± 73.9	601.2 ^b^ ± 23.3	423.3 ^a^ ± 35.2	595.2 ^b^ ± 72.2
methyl 2-hydroxy-4-methylpentanoate	4.1 ^c^ ± 0.4	2.3 ^b^ ± 0.4	2.1 ^b^ ± 0.1	2.2 ^b^ ± 0.2	1.1 ^a^ ± 0.1	1.2 ^a^ ± 0.1	1.0 ^a^ ± 0.1
ethyl 3-hydroxybutyrate	104.6 ^b^ ± 2.5	43.5 ^a^ ± 1.9	43.8 ^a^ ± 2.7	46.2 ^a^ ± 2.3	49.0 ^a^ ± 2.4	49.6 ^a^ ± 3.7	48.3 ^a^ ± 10.5
ethyl 2-hydroxy-4-methylpentanoate	28.9 ^a^ ± 4.7	80.3 ^a^ ± 42.8	58.8 ^a^ ± 12.8	84.1 ^a^ ± 43.4	33.7 ^a^ ± 1.8	28.3 ^a^ ± 1.9	34.8 ^a^ ± 12.3
diethyl malonate	2.5 ^a^ ± 0.1	6.7 ^b^ ± 0.0	7.0 ^c^ ± 0.2	6.7 ^b^ ± 0.2	2.6 ^a^ ± 0.1	2.6 ^a^ ± 0.1	2.7 ^a^ ± 0.2
ethyl decanoate	431.8 ^e^ ± 4.7	78.0 ^c^ ± 9.3	30.7 ^a^ ± 6.6	29.5 ^a^ ± 8.8	101.9 ^d^ ± 12.1	54.8 ^b^ ± 11.3	68.9 ^b,c^ ± 15.1
ethyl 4-hydroxybutyrate	278.2 ^c^ ± 1.7	24.0 ^a^ ± 0.9	22.4 ^a^ ± 2.5	21.8 ^a^ ± 0.4	56.7 ^b^ ± 6.1	57.5 ^b^ ± 1.8	55.3 ^b^ ± 2.7
1,3-propanediol diacetate	50.0 ^c^ ± 4.2	2.2 ^a^ ± 0.3	2.2 ^a^ ± 0.2	2.1 ^a^ ± 0.1	10.7 ^b^ ± 0.8	10.1 ^b^ ± 0.2	9.5 ^b^ ± 0.1
ethyl 3-hydroxyhexanoate	2.7 ^c^ ± 0.1	2.4 ^b^ ± 0.1	2.3 ^b^ ± 0.1	2.2 ^b^ ± 0.1	1.5 ^a^ ± 0.1	1.5 ^a^ ± 0.1	1.5 ^a^ ± 0.1
ethyl succinate	3288.8 ^a^ ± 996.6	10398.5 ^c^ ± 990.9	10385.3 ^c^ ± 913.3	9051.2 ^a,c^ ± 1246.3	7100.7 ^a^ ± 1214.6	8395.3 ^a,c^ ± 475.6	7262.3 ^a^ ± 808.8
ethyl glutarate	21.7 ^a^ ± 1.8	98.4 ^c^ ± 0.6	95.7 ^c^ ± 7.8	93.6 ^c^ ± 6.6	47.9 ^b^ ± 3.8	48.2 ^b^ ± 4.9	43.9 ^b^ ± 6.8
**C6 alcohols**							
1-hexanol	1808.0 ^a^ ± 57.2	1973.1 ^a^ ± 211.3	2017.6 ^a^ ± 137.5	2058.1 ^a^ ± 61.2	1920.5 ^a^ ± 239.8	2000.3 ^a^ ± 105.0	1669.0 ^a^ ± 61.3
*cis*-3-hexen-1-ol	216.4 ^a^ ± 4.1	238.8 ^a^ ± 31.7	243.4 ^a^ ± 18.0	243.5 ^a^ ± 12.0	227.8 ^a^ ± 23.4	247.5 ^a^ ± 12.9	218.7 ^a^ ± 21.3
*trans*-2-hexen-1-ol	5.0 ^b^ ± 0.3	3.0 ^a^ ± 0.6	3.6 ^a^ ± 0.7	3.0 ^a^ ± 0.2	7.9 ^c^ ± 0.4	8.0 ^c^ ± 0.5	8.2 ^c^ ± 1.3
*cis*-2-hexen-1-ol	34.4 ^a^ ± 3.0	32.3 ^a^ ± 3.5	30.9 ^a^ ± 2.2	29.9 ^a^ ± 2.2	28.4 ^a^ ± 3.0	29.3 ^a^ ± 2.9	28.7 ^a^ ± 4.0
**Benzenic compounds**							
benzaldehyde	34.0 ^a^ ± 1.1	35.5 ^a^ ± 4.1	37.9 ^a^ ± 5.1	35.8 ^a^ ± 2.1	39.9 ^a^ ± 3.4	42.3 ^a^ ± 2.8	37.0 ^a^ ± 1.9
2-phenylethyl acetate	582.0 ^c^ ± 2.8	21.5 ^a^ ± 1.5	23.0 ^a^ ± 1.4	20.7 ^a^ ± 0.7	178.0 ^b^ ± 13.4	181.4 ^b^ ± 5.8	175.8 ^b^ ± 1.7
guaiacol	10.1 ^c^ ± 0.4	8.3 ^b,c^ ± 1.4	8.1 ^b,c^ ± 1.4	6.8 ^a,b^ ± 1.3	5.3 ^a^ ± 0.7	7.3 ^a,b^ ± 0.4	6.2 ^a,b^ ±0.8
benzyl alcohol	221.9 ^a^ ± 4.3	263.0 ^a^ ± 96.1	309.8 ^a^ ± 99.9	186.5 ^a^ ± 22.5	251.5 ^a^ ± 19.6	272.9 ^a^ ± 45.4	286.9 ^a^ ± 67.3
phenyl ethyl alcohol	8384.9 ^a^ ± 802.5	7914.4 ^a^ ± 3082.9	9311.7 ^a^ ± 1997.6	9234.5 ^a^ ± 3182.7	9166.7 ^a^ ± 933.2	8556.5 ^a^ ± 1506.6	8724.8 ^a^ ± 1375.6
4-vinylguaiacol	564.9 ^b^ ± 49.3	624.2 ^b^ ± 66.5	509.5 ^b^ ± 14.6	550.7 ^b^ ± 96.6	330.6 ^a^ ± 77.4	373.5 ^a^ ± 34.0	402.5 ^a^ ± 35.6
**Terpenes**							
*trans*-linalool oxide	2.6 ^b^ ± 0.3	7.4 ^c^ ± 0.1	7.5 ^c^ ± 0.2	7.5 ^c^ ± 0,1	1.9 ^a^ ± 0.2	1.9 ^a^ ± 0.1	1.8 ^a^ ± 0.2
*cis*-linalool oxide	1.9 ^a^ ± 0.3	4.3 ^b^ ± 0.2	4.2 ^b^ ± 0.2	3.9 ^b^ ± 0.4	1.4 ^a^ ± 0.1	1.4 ^a^ ± 0.1	1.4 ^a^ ± 0.1
linalool	5.0 ^c^ ± 0.4	0.6 ^a^ ± 0.2	0.5 ^a^ ± 0.1	0.6 ^a^ ± 0.1	3.8 ^b^ ± 0.4	3.6 ^b^ ± 0.0	3.5 ^b^ ± 0.2
hotrienol	2.4 ^a,b^ ± 0.0	3.1 ^b^ ± 0.8	2.8 ^b^ ± 0.8	3.0 ^b^ ± 0.3	1.3 ^a^ ± 0.4	1.4 ^a^ ± 0.2	1.3 ^a^ ± 0.3
α-terpineol	6.0 ^a^ ± 0.1	29.8 ^b^ ± 4.6	27.2 ^b^ ± 3.6	27.7 ^b^ ± 3.8	11.8 ^a^ ± 2.0	12.4 ^a^ ± 0.3	11.9 ^a^ ± 3.0
citronellol	3.4 ^b^ ± 0.2	1.7 ^a^ ± 0.4	1.6 ^a^ ± 0.2	1.5 ^a^ ± 0.1	1.5 ^a^ ± 0.1	1.5 ^a^ ± 0.0	1.5 ^a^ ± 0.2
*trans*-geraniol	1.7 ^a^ ± 0.3	1.3 ^a^ ± 0.2	1.5 ^a^ ± 0.3	1.3 ^a^ ± 0.1	1.2 ^a^ ± 0.2	1.4 ^a^ ± 0.2	1.3 ^a^ ± 0.1
hydroxylinalool	3.4 ^c^ ± 0.1	2.1 ^a,b^ ± 0.2	1.5 ^a^ ± 0.2	1.6 ^a^ ± 0.1	2.6 ^b^ ± 0.3	2.7 ^b^ ± 0.2	2.7 ^b^ ± 0.6
geranic acid	22.1 ^c^ ± 1.2	10.1 ^b^ ± 1.4	8.6 ^b,a^ ± 0.3	7.3 ^a^ ± 0.3	9.9 ^b^ ± 0.4	6.7 ^a^ ± 0.7	7.1 ^a^ ± 0.6
**C13-norisoprenoids**							
vitispirane	0.7 ^a^ ± 0.1	6.9 ^c^ ± 0.8	6.3 ^b,c^ ± 0.7	5.8 ^b^ ± 0.5	0.6 ^a^ ± 0.0	0.6 ^a^ ± 0.0	0.7 ^a^ ± 0.1
β-damascenone	13.4 ^b^ ± 0.6	7.3 ^a^ ± 0.7	6.7 ^a^ ± 0.7	6.1 ^a^ ± 0.5	6.4 ^a^ ± 1.0	5.3 ^a^ ± 0.8	5.9 ^a^ ± 0.1
α-ionone	1.1 ^a^ ± 0.3	1.0 ^a^ ± 0.2	0.9 ^a^ ± 0.3	1.0 ^a^ ± 0.1	0.8 ^a^ ± 0.3	0.6 ^a^ ± 0.3	0.7 ^a^ ± 0.2
TDN	9.7 ^a^ ± 0.2	24.7 ^b^ ± 4.9	23.3 ^b^ ± 7.4	25.7 ^b^ ± 2.4	8.8 ^a^ ± 2.5	9.2 ^a^ ± 1.7	9.9 ^a^ ± 2.3
3-oxo-α-ionol	82.0 ^b^ ± 10.9	45.0 ^a^ ± 3.5	45.1 ^a^ ± 3.0	40.6 ^a^ ± 4.8	43.2 ^a^ ± 5.0	41.1 ^a^ ± 2.8	41.2 ^a^ ± 3.6
**Lactones**							
γ-butyrolactone	12.7 ^a^ ± 2.1	11.7 ^a^ ± 12.2	7.9 ^a^ ± 4.0	13.9 ^a^ ± 14.6	15.4 ^a^ ± 2.0	16.0 ^a^ ± 2.6	13.4 ^a^ ± 1.4
γ-caprolactone	1.4 ^a^ ± 0.1	2.4 ^b^ ± 0.3	2.3 ^b^ ± 0.3	2.2 ^b^ ± 0.1	2.2 ^b^ ± 0.1	2.3 ^b^ ± 0.1	2.4 ^b^ ± 0.1
γ-nonalactone	12.4 ^a^ ± 0.7	18.9 ^c^ ± 0.9	15.8 ^b^ ± 2.2	17.6 ^b,c^ ± 1.2	11.2 ^a^ ± 1.2	9.9 ^a^ ± 0.5	10.1 ^a^ ± 0.7
pantolactone	0.03 ^a^ ± 0.00	0.08 ^b^ ± 0.01	0.08 ^b^ ± 0.01	0.07 ^b^ ± 0.00	0.03 ^c^ ± 0.00	0.03 ^c^ ± 0.00	0.03 ^c^ ± 0.00
γ-decalactone	512.9 ^a^ ± 29.3	817.9 ^b^ ± 84.0	775.1 ^b^ ± 52.4	776.3 ^b^ ± 37.0	484.5 ^a^ ± 52.3	434.7 ^a^ ± 5.2	446.7 ^a^ ± 32.0
**Furan and pyran compounds**							
furfural	23.9 ^a^ ± 2.1	156.4 ^b^ ± 19.4	154.6 ^b^ ± 21.6	167.2 ^b^ ± 9.3	21.3 ^a^ ± 11.8	18.0 ^a^ ± 7.9	11.9 ^a^ ± 2.9
furanyl ethanone	4.0 ^a^ ± 0.4	15.1 ^b^ ± 1.3	15.3 ^b^ ± 1.5	16.2 ^b^ ± 0.5	3.9 ^a^ ± 0.3	4.0 ^a^ ± 0.3	4.3 ^a^ ± 0.8
ethyl 2-furoate	13.9 ^a^ ± 0.2	94.7 ^c^ ± 2.3	95.8 ^c^ ± 3.5	97.0 ^c^ ± 3.2	32.3 ^b^ ± 1.3	33.9 ^b^ ± 3.1	29.6 ^b^ ± 0.9
5-ethoxymethylfurfural	88.6 ^a^ ± 2.0	198.3 ^c^ ± 13.6	192.9 ^c^ ± 18.2	192.7 ^c^ ± 18.0	130.8 ^b^ ± 8.0	142.2 ^b^ ± 4.8	121.0 ^b^ ± 11.3
furaneol	1.4 ^a^ ± 0.2	3.5 ^b^ ± 0.5	3.4 ^b^ ± 0.6	2.9 ^a,b^ ± 0.5	2.2 ^a^ ± 0.2	2.6 ^a^ ± 0.1	2.6 ^a^ ± 0.1
maltol	29.1 ^a^ ± 2.0	25.5 ^a^ ± 5.2	22.9 ^a^ ± 2.2	25.2 ^a^ ± 7.6	31.7 ^a^ ± 10.8	23.4 ^a^ ± 6.8	19.5 ^a^ ± 5.9
5-hydroxymethylfurfural	4.4 ^a^ ± 0.1	47.0 ^b^ ± 5.5	47.0 ^b^ ± 4.8	44.4 ^b^ ± 3.0	7.8 ^a^ ± 0.8	7.3 ^a^ ± 0.9	8.5 ^a^ ± 0.6
**Acids**							
butyric acid	226.9 ^a^ ± 5.7	385.6 ^a^ ± 242.7	272.9 ^a^ ± 112.0	231.4 ^a^ ± 159.2	221.9 ^a^ ± 6.7	317.2 ^a^ ± 196.2	234.4 ^a^ ± 44.0
hexanoic acid	5847.9 ^a^ ± 300.0	5544.9 ^a^ ± 276.8	5443.1 ^a^ ± 277.0	5176.0 ^a^ ± 180.5	5114.5 ^a^ ± 1064.0	5617.3 ^a^ ± 198.6	5004.1 ^a^ ± 945.3
octanoic acid	4516.3 ^a^ ± 155.4	4339.8 ^a^ ± 1649.4	3073.2 ^a^ ± 1210.7	3191.2 ^a^ ± 1121.1	4956.1 ^a^ ± 782.6	4409.9 ^a^ ± 1288.5	4984.3 ^a^ ± 1361.7
decanoic acid	745.1 ^b^ ± 13.7	496.7 ^a,b^ ± 36.5	464.2 ^a,b^ ± 82.5	379.5 ^a^ ± 128.4	747.3 ^b^ ± 193.2	538.2 ^a,b^ ± 148.5	637.4 ^a,b^ ± 82.2
methyl propanoic acid	4.3 ^a^ ± 0.1	5.0 ^a^ ± 4.1	2.6 ^a^ ± 1.0	2.7 ^a^ ± 0.6	3.1 ^a^ ± 0.3	3.3 ^a^ ± 0.6	4.2 ^a^ ± 0.9
**Sulfur compounds**							
2-mercaptoethanol	1.7 ^c^ ± 0.3	1.2 ^b^ ± 0.3	1.1 ^b^ ± 0.1	0.9 ^a,b^ ± 0.1	0.6 ^a^ ± 0.1	0.9 ^a,b^ ± 0.1	0.9 ^a,b^ ± 0.1
2-methyl-dihydro-thiophen-3-one	100.0 ^b^ ±5.6	43.3 ^a^ ± 2.3	43.7 ^a^ ± 5.0	46.9 ^a^ ± 1.8	44.1 ^a^ ± 4.1	46.8 ^a^ ± 4.8	45.8 ^a^ ± 11.9
ethyl 3-methylthiopropanoate	0.7 ^b^ ± 0.0	0.9 ^c^ ± 0.0	0.9 ^c^ ± 0.1	0.9 ^c^ ± 0.0	0.5 ^a^ ± 0.0	0.5 ^a^ ± 0.0	0.5 ^a^ ± 0.1
methionol	65.4 ^b^ ± 0.4	69.5 ^b^ ± 6.6	61.6 ^b^ ± 2.7	65.7 ^b^ ± 2.5	48.5 ^a^ ± 2.6	52.0 ^a^ ± 4.9	53.5 ^a^ ± 6.9
3-methylthiopropanoic acid	7.5 ^b^ ± 2.4	2.5 ^a^ ± 0.5	2.4 ^a^ ± 0.2	2.5 ^a^ ± 0.2	1.8 ^a^ ± 0.1	2.3 ^a^ ± 1.2	2.9 ^a^ ± 0.5

^a,b,c,d,e^ Different letters in the same row mean significant differences (α = 0.05) according to the test of Student–Newman–Keuls. nd: not detected.

## Data Availability

Not available.

## Sample Availability

Not available.
